# Symptoms and objective signs of peripheral sensory neuropathy in POTS and correlations to gastrointestinal symptoms

**DOI:** 10.1371/journal.pone.0327549

**Published:** 2025-07-03

**Authors:** Linnéa Ekman, Hanna Tufvesson, Elisabet Englund, Lars B. Dahlin, Bodil Ohlsson

**Affiliations:** 1 Department of Translational Medicine, Division of Hand Surgery, Lund University, Malmö, Sweden; 2 Department of Clinical Sciences, Division of Pathology, Lund University, Lund, Sweden; 3 Department of Clinical Sciences, Lund University, Malmö, Sweden; 4 Department of Gastroenterology, Skåne University Hospital, Malmö, Sweden; 5 Department of Hand Surgery, Skåne University Hospital, Malmö, Sweden; 6 Department of Biomedical and Clinical Sciences, Linköping University, Linköping, Sweden; 7 Department of Internal Medicine, Skåne University Hospital, Malmö, Sweden; Weill Cornell Medicine-Qatar, QATAR

## Abstract

**Aim:**

Postural orthostatic tachycardia syndrome (POTS) is a disorder with cardiovascular autonomic dysfunction where multiple and variable symptoms are common, including those from the peripheral and enteric nervous systems. We aimed to investigate subjective and objective signs of small and large fiber neuropathy in a Swedish POTS cohort compared with healthy controls. Secondly, we wanted to examine potential associations between gastrointestinal symptoms and neuropathy signs in POTS.

**Methods:**

Forty-three patients with POTS (93% female) and 54 healthy controls (76% female) were included in the study. All participants completed a questionnaire including modified neuropathy symptoms score (NSS) and irritable bowel syndrome severity scoring system (IBS-SSS) for gastrointestinal evaluation. Small nerve fibers were investigated by assessing the intraepidermal nerve fiber density (IENFD). Large nerve fiber function was examined through pinprick and vibration perception thresholds (VPTs), using neurothesiometry and multi-frequency vibrometry (MFV). The patients were classified as “High NSS” and “High IBS-SSS” if their total NSS vs. IBS-SSS were above median levels in POTS.

**Results:**

Peripheral sensory and gastrointestinal symptoms were more prevalent and severe in POTS than in controls. Median VPTs were normal and IENFDs were comparable between POTS and controls (2.26 [1.62–3.08] vs. 1.63 [0.73–2.68] fibers/mm; p = 0.108). The patients with “high NSS” had slightly higher VPTs measured by MFV, although within normal ranges, compared to patients with “low NSS”. The patients within the “high IBS-SSS” group had higher NSS (18.0 [14.3–22.8] vs. 11.0 [4.0–15.0]; p = 0.002) compared to patients with low total IBS-SSS.

**Conclusion:**

Symptoms of peripheral and enteric neuropathy are common in POTS but no solid evidence was found regarding functional or morphological signs of small or large fiber neuropathy. Neuropathic and gastrointestinal symptoms were closely associated within POTS.

## 1. Introduction

Postural orthostatic tachycardia syndrome (POTS) is a disorder characterized by debilitating orthostatic symptoms and pulse acceleration upon standing with no significant drop in blood pressure [1]. The mechanisms behind POTS are probably multifactorial and still not fully elucidated [2]. One mechanistic subtype is the neuropathic POTS, where the suggested pathophysiology involves underlying small nerve fiber neuropathy (SFN), characterized by degeneration and/or dysfunction of thinly myelinated Aδ-fibers and unmyelinated C-fibers [3]. Both the somatic and the autonomic peripheral nervous systems, including the enteric nerves, can be affected by SFN [4,5], but the accessibility to peripheral nerves is superior to enteric nerves [5]. Patients with POTS may experience symptoms consistent with peripheral sensory small and large nerve fiber neuropathy [6]. Previous studies have reported objective signs of SFN in some POTS patients, where assessments of intraepidermal nerve fiber density (IENFD) in skin biopsies have shown reduced nerve fiber density in up to 56% of the POTS population [5,7–10]. Moreover, post-ganglionic sudomotor dysfunction, measured by quantitative sudomotor axon reflex test (QSART), reflecting partial sympathetic denervation is common in various autonomic neuropathies [11] and is present in some POTS patients [9,12–14]. We have previously shown that gastrointestinal symptoms were common and moderately severe in POTS, resembling the symptomatology found in irritable bowel syndrome (IBS), as well as in gastroparesis [15]. Additionally, we have published a case report where two patients with known enteric neuropathy also presented with symptoms of peripheral neuropathy, and reduced IENFD. One of the patients also fulfilled criteria for POTS [5].

Regarding peripheral neuropathic involvement, few studies have compared patients with POTS to healthy matched controls [7]. Thereto, of the studies that have shown reduced IENFD in POTS, three were retrospective [8–10]. Thus, no symptom evaluation was performed within temporal proximity to the biopsy sampling. In the study by Gibbons et al [7], POTS patients with objective signs of post-ganglionic sudomotor dysfunction reported more gastrointestinal symptoms, suggesting a more widespread neuropathy. However, symptoms of peripheral sensory neuropathy were not assessed in that study. To our knowledge, the only previous study that has examined large nerve fiber function in POTS was conducted retrospectively [10].

Therefore, we aimed to assess symptoms and objective signs of peripheral small and large fiber neuropathy in POTS to determine any correlations between the severity of symptoms and the extent of objective neuropathy. Additionally, since patients with POTS also experience symptoms that may originate from the enteric nervous system, we aimed to examine whether gastrointestinal symptoms were associated with subjective or objective signs of peripheral neuropathy, reflecting a more widespread neuropathy.

## 2. Materials & methods

### 2.1. Ethical statement

The present study was performed in accordance with the Declaration of Helsinki and approved by the Ethics Review Board at Uppsala University, permission no. 2020−02432 (approval date 26/08/2020) and 2021−00049 (approval date 11/02/2021). Written informed consent was obtained from all study participants.

### 2.2. Study design

In the present cross-sectional study, participants were recruited at the Department of Gastroenterology and the Department of Hand Surgery, Skåne University Hospital, Malmö, Sweden, between the 1:st of October 2020 and 31:st of May 2023. Invitations, including study information, were sent by regular mail to patients with a previously described and confirmed diagnosis of POTS at the Department of Cardiology at Skåne University Hospital, Malmö, Sweden [16]. One of the authors (HT) contacted the participants by telephone, to give further information and book a visit. At the same time, healthy control participants were recruited among hospital staff and students. The study was conducted in two study sessions. During the first study visit, the participants were asked to fill in study questionnaires, and a clinical examination was performed. Large sensory nerve fiber function [17] was examined with routine clinical tests commonly used for neuropathy screening: 10 g monofilament test for pinprick sense, and neurothesiometry for vibrotactile sense [18]. A second study visit was then booked within a couple of weeks, where an additional, more comprehensive, examination of large sensory nerve fibers was performed with multi-frequency vibrometry (MFV), and two skin biopsies were obtained from the distal leg to determine IENFD and small nerve fiber neuropathy.

### 2.3. Study population

#### 2.3.1. Patients with POTS.

Study participants (n = 43) were recruited from the Syncope Study of Unselected Population in Malmö (SYSTEMA) cohort. This cohort involves over 3000 patients investigated for syncope and severe orthostatic intolerance at the Skåne University Hospital in Malmö, Sweden, and was conducted between 2008 and 2021 [19]. All patients of the cohort underwent cardiovascular autonomic tests with head-up tilt testing with continuous hemodynamic monitoring, as well as other cardiac tests, including ambulatory electrocardiogram or 24-hour ambulatory blood pressure monitoring, when appropriate. The head-up tilt testing protocol included supine rest for 10 min preceding table elevation to 60–70° for 20 min [20]. The diagnosis of POTS was defined as symptoms of orthostatic intolerance lasting for ≥ 3 months associated with a pathological head-up tilt test showing a heart rate (HR) increase of ≥ 30 beats per minute (bpm) or HR ≥ 120 bpm, with no significant drop in blood pressure [21]. All patients fulfilled the diagnostic criteria for POTS. The recruitment process from this cohort has been described in detail previously [15], and is summarized in a flow chart in supplementary figure 1. Inclusion criteria were ages between 18–70 years and the ability to fully understand the study information.

#### 2.3.2. Healthy controls.

Healthy control participants (n = 54) were recruited among hospital staff, medical students, and their relatives at Skåne University Hospital, Malmö, through personal invitation and advertisement. The controls were not allowed to have any current chronic or acute illness. Intake of multivitamins and hormonal contraceptive medicines was accepted, but otherwise, only temporary use of medications, such as seasonal allergy medicines, was allowed.

### 2.4. Questionnaires

All study participants were asked to complete a questionnaire, including sociodemographic factors, lifestyle habits, previous and current illnesses, family history, and current pharmacological treatment. Furthermore, two validated symptom scores, described below, were used to assess subjective symptoms of peripheral neuropathy and gastrointestinal symptoms.

#### 2.4.1. Neuropathy Symptom Score (NSS).

A modified version of the validated Neuropathy Symptom Score (NSS) was used to assess subjective signs of peripheral neuropathy in the hands and feet [22]. The symptoms assessed are numbness, warmth/cold sensation, paresthesia, burning sensation, radiating pain, aching pain, and nocturnal exacerbation by sheets. The participants scored the symptoms as 0 = never, 1 = sometimes, 2 = often, and 3 = nocturnal. Scores for all questions regarding both hands and feet were added to produce a composite total score (maximum 42 points). If the respondent answered that symptoms occurred both often (2 points) and nocturnally (3 points), the highest point was used in the composite score.

#### 2.4.2. The irritable bowel syndrome severity scoring system (IBS-SSS).

While most of the patients in the present study did not have an official IBS diagnosis, the comorbidity with IBS is high in POTS [6,15]. We have previously used this tool for assessment of gastrointestinal symptoms in this very POTS cohort, revealing a wide span of gastrointestinal symptom severity [15]. The IBS-SSS questionnaire is a validated scoring system used in IBS, consisting of four items regarding abdominal pain, abdominal distension, satisfaction with bowel habits, and the impact of bowel habits on daily life. The items are rated on a visual analog scale from zero to 100 millimeters, where 100 mm indicates very severe symptoms. In addition, one question asks the number of days of abdominal pain in the last 10 days. Scores from these questions are added together, producing a total IBS-SSS, with a maximum score of 500. Scores between 75 and 174 suggest mild IBS, 175–299 suggest moderate IBS, and ≥ 300 suggest severe IBS [23].

### 2.5. Clinical examinations

Patients with POTS were clinically examined with heart-, lung-, abdominal- and routine neurological status [24]. In all participants, neurological sensory testing for large sensory nerve fibers and mechanoreceptors was performed distally at the feet and leg with touch, by 10 g monofilament (Bailey Instruments, Manchester, UK), and by neurothesiometry. Examination with Horwell Neurothesiometer (Scientific Laboratory Supplies, Nottingham, UK) was performed with the participants in a supine position, to assess vibration perception thresholds (VPTs) at the bony prominence of the medial malleolus and the tip of the big toe bilaterally. The vibrating frequency was preset to 50–60 Hz on this device and the vibratory signal was increased slowly until the participant sensed the vibration, measured in volts (V). Three measurements were performed at each site, and a mean value was calculated. Phalen’s and Tinel’s tests were performed, as well as anamnestic symptoms of median nerve affection were asked for, to exclude carpal tunnel syndrome in all participants [25]. The current weight and height were obtained. All clinical examinations were performed by an internal medicine specialist (HT).

### 2.6. Multi-frequency vibrometry

Vibration perception thresholds were further examined in patients with POTS using the VibroSense Meter® II device (VibroSense Dynamics AB, Malmö, Sweden) on the index and little finger of the right hand as well as on the head of the first metatarsal bone of the right foot. A full program, i.e., seven frequencies (4, 8, 16, 32, 64, 125, and 250 Hz), was used for examining VPTs in the foot. For VPT assessment in the index and little finger, a screening procedure was applied, consisting of only three frequencies (32, 125, and 250 Hz). A detailed description of the method has been presented previously [26]. The results were expressed as Z-scores, based on data obtained from previous studies with healthy age- and sex-matched controls [26]. Thus, none of the healthy controls recruited for the present study were examined through MFV.

### 2.7. Skin biopsy procedure and evaluation of intraepidermal nerve fiber density

A skin punch biopsy was obtained from the distal right leg, 10 cm proximal to the lateral malleolus, using a 3 mm disposable circular needle during local anesthesia (0.5 ml Carbocain® 10 mg/ml) (LD). The skin defect was closed with one Ethilon 4−0 suture (Ethicon®, Raritan, USA) and covered by a small dressing allowing free activities. Biopsy samples were immediately fixed in a 4% buffered formaldehyde solution for at least 24 hours before dehydration and paraffin embedding.

Thereafter, the paraffin-embedded blocks were sectioned at 5 µm and immunohistochemically stained with a rabbit polyclonal Protein Gene Product (PGP) 9.5 antibody (Cell Marque, Rocklin, USA, dilution 1:3000). The IENFD, expressed as the number of fibers per millimeter of epidermal length, was manually assessed in each specimen by one of the authors (LE). Nerve fibers were counted as previously validated, and described in a large normal material [27]. The observer was blinded to the characteristics of the participants when assessing IENFD. Inter- and intra-observer reliability was assessed with good results in a previous study by the same author (LE) using the same methods as here [28]. For choice of methods, please see Discussion.

### 2.8. Statistical analyses

Statistical analyses were performed in SPSS, version 28. Data are presented as median and interquartile range (IQR) or numbers and percentages (N, %). Data on VPTs assessed with MFV are presented as average Z-scores for each measuring point (index finger, little finger, first metatarsal head). Differences between patients and controls were compared using the non-parametric Mann-Whitney *U* test. Fisher’s exact test was used for dichotomous variables. P < 0.05 was considered statistically significant.

## 3. Results

### 3.1. Basic characteristics

A total of 43 patients (age 31 [26–41] years) and 54 controls (age 38 [30–46] years; p = 0.010) were included in the study. There was a higher proportion of female sex (n = 40; 93.0%) in the patient group in comparison to the control group (n = 41; 75.6%; p = 0.029). There was no difference in BMI between patients (24.2 [21.3–27.0] kg/m^2^) and controls (22.7 [20.8–25.3] kg/m^2^); p = 0.294). Most of the patients (74.4%) and the controls (72.2%) had never smoked (p = 0.528). The vast majority (88.4%) of the patients consumed less than one standard glass of alcohol per week, which was significantly fewer than in the control group (31.5% < 1 glass; p < 0.001).

Besides POTS, the most common self-reported comorbidities among patients were Ehlers-Danlos syndrome (n = 12; 27.9%), IBS (n = 12; 27.9%), asthma (n = 8; 18.6%), migraine (n = 7; 16.3%), neuropsychiatric disorders (n = 6; 14.0%), endometriosis (n = 5; 11.6%), and myalgic encephalomyelitis (n = 5; 11.6%). In the control group, seasonal allergies were found in four (7.4%) participants. The most common drugs in the POTS patients were antihypotensive drugs (n = 17; 39.5%), ivabradine (n = 16; 37.2%), beta-blockers (n = 13; 30.2%), histamine H1 blockers (n = 13; 30.2%), and combined hormonal contraceptives (n = 8; 18.6%). A full list of current drug treatments in this POTS cohort has previously been published [15]. Sporadic users of anti-allergy treatment, contraceptives, and multivitamins were found among healthy controls.

### 3.2. Questionnaires

#### 3.2.1. Neuropathy Symptom Score (NSS).

Four (7.4%) of the 54 control participants and 36 (83.7%) of the 43 patients with POTS reported one or more variables on the NSS to occur often or nocturnally in hands or feet. Significant differences in total NSS were found between patients and controls where the median composite score for patients was 14.5 (6.0–21.3) compared to 0.0 (0.0–2.0) in the control group (p < 0.001; Table 1).

**Table 1:** Clinical characteristics and subjective and objective signs of sensory nerve fiber neuropathy in control participants and patients with POTS.

	Controls N = 54	POTS N = 43	P-value
**Age** (years)	38 (30–46)	31 (26–41)	**0.010**
**Female** (%)	41 (75.6%)	40 (93.0%)	**0.029**
**Monofilament, normal** (%)	46 (90.2%)^d^	39 (95.1%)^b^	0.455
**Neurothesiometer** (V)			
**Right big toe**	5.8 (4.8–7.7)^a^	4.9 (4.0–7.1)^a^	0.065
**Left big toe**	5.8 (4.2–7.9)^a^	5.1 (3.3–6.7)^a^	0.085
**Right lateral malleolous**	7.3 (5.8–9.5)^a^	7.3 (5.7–9.2)^a^	0.810
**Left lateral malleolous**	7.7 (6.2–9.7)^a^	6.5 (4.7–8.5)^a^	**0.016**
**IBS-SSS total**	11.0 (0.0–46.8)	212.5 (134.5–318.8)^c^	**<0.001**
**NSS total**	0.0 (0.0–2.0)	14.5 (6.0–21−3)^a^	**<0.001**
**Multi-frequency vibrometry** (average Z-score)			
**Hand**			
Index finger	n/a	0.3 (−0.4–1.1)^d^	
Little finger	n/a	0.6 (−0.3–1.1)^d^	
**Foot**	n/a	0.1 (−0.4–0.7)^d^	
**IENFD** (fibers/mm)	1.63 (0.73–2.68)^e^	2.26 (1.62–3.08)^d^	0.108

Values presented as number (percent) or median (interquartile range). Comparative analyses were performed with Fisher’s Exact test or Mann Whitney-U test. A p-value < 0.05 was considered statistically significant. IBS-SSS: Irritable Bowel Syndrome – Severity Scoring System [23]; NSS: Neuropathy Symptom Score [22]; IENFD: Intraepidermal Nerve Fiber Density. n/a = not applicable

^a^1 missing value.

^b^2 missing values.

^c^3 missing values.

^d^4 missing values.

^e^13 control participants were examined.

The most frequently reported symptoms in POTS patients were “Abnormal sensation to heat or cold”, reported to occur often or nocturnally by 25 (59.5%) and 28 (66.7%) patients in hands and feet, respectively, and “Sensation of pins and needles”, reported to occur often or nocturnally by 20 (47.6%) and 19 (45.2%) patients in hands and feet, respectively. All items on the NSS questionnaire were reported to occur more or less often in the POTS cohort. There was no difference in the distribution of neuropathic symptoms between hands and feet in POTS (S1 Table).

#### 3.2.2. The irritable bowel syndrome severity scoring system (IBS-SSS).

The median total IBS-SSS score was 213 (135–319) in POTS compared to 11 (0–47) in healthy controls (p < 0.001, Table 1).

### 3.3. Clinical examinations

Clinical routine neurological status was generally normal and without any objective signs of severe neurological disorder in POTS. Abnormal signs of motor neuropathy were observed in sporadic cases: walking test (n = 2; 4.7%), walking on toes (n = 3; 7.0%), walking on heels (n = 5; 11.6%), finger strength (n = 2; 4.7%), shoulder abduction (n = 2; 4.8%), and leg raises (n = 6; 14.0%). In all these cases, general weakness was observed clinically, however, the clinical examination did not raise any suspicion of neurological disorders in any of these cases. Five of the POTS patients were using wheelchairs due to orthostatic intolerance or general muscle weakness (clinical observation). Brachial, patellar, and Achilles tendon reflexes were tested in all patients. In a handful of patients, reflexes were difficult to elicit in one or two reflex sites. However, there were no patients who had absent reflexes in all of the tested sites.

No differences were found between patients and controls regarding pinprick sensation with monofilament testing (Table 1). The VPTs measured by neurothesiometer were lower in the left medial malleolus in patients compared with controls, although within the normal range (Table 1). Four (9.3%) of the patients experienced unilaterally mildly decreased or increased sensitivity in the arms, and five (11.6%) experienced unilaterally mildly decreased or increased sensitivity in the legs upon touching. None of these patients had abnormal results from neurothesiometer or monofilament testing.

Phalen’s test was positive in three patients and three controls, whereas Tinel’s test was positive in seven of the patients and three controls. One POTS patient fulfilled the criteria for carpal tunnel syndrome after re-evaluation at the Department of Hand Surgery and the other two of the patients with positive Phalen’s test were already operated on for carpal tunnel syndrome with no effect on symptoms. All of the POTS patients with positive Phalen’s test reported various neuropathic symptoms graded with the NSS as often in both hands *and* feet. None of the control participants with positive Phalen’s test had any subjective symptoms of neuropathy measured on the NSS. One control participant had clinical signs of ulnar nerve entrapment, which was confirmed after re-evaluation at the Department of Hand Surgery.

### 3.4. Multi-frequency vibrometry

Average Z-scores for VPTs assessed through MFV are presented for patients in Table 1. Pathological measures (average Z-score >1.65) were found in the feet of three patients, as well as in the index and/or little fingers of five patients. Among the patients with a vibrometry Z-score >1.65 in the index and/or little fingers, only one showed a positive Tinel’s test. None of these patients presented with a positive Phalen’s test.

### 3.5. Intraepidermal nerve fiber density

**Fig 1:**
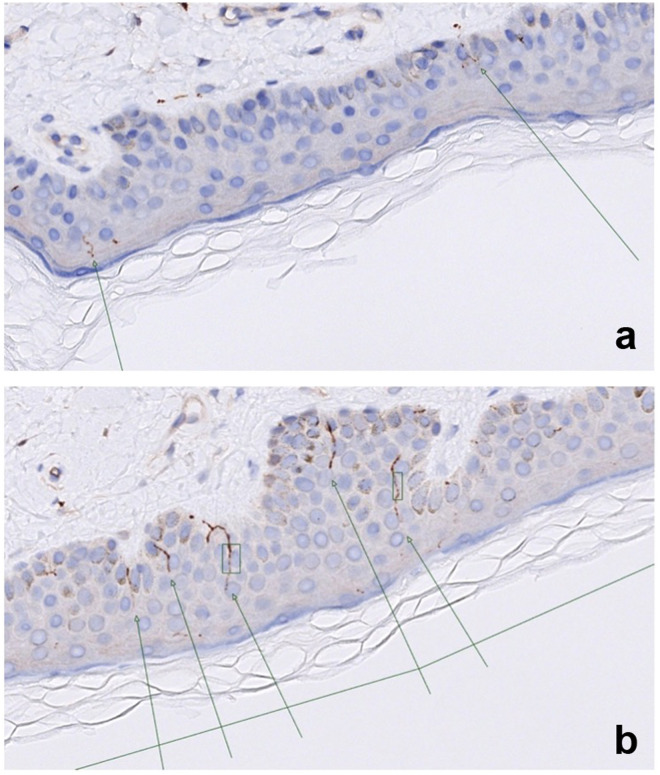
Intraepidermal nerve fibers. Intraepidermal nerve fibers in 5 µm skin section on a 50X magnification in a) a patient with low IENFD and b) high IENFD. Nerve fibers are indicated by arrows. IENFD: Intraepidermal nerve fiber density.

Thirty-nine (90.4%) of the patients with POTS and 13 (24.1%) of the healthy control participants underwent skin punch biopsy. In general, skin biopsies were well tolerated in both POTS and controls. A mild and local skin infection occurred in one patient who consequently received antibiotic therapy with no residual complications. Regarding the subgroup of POTS patients and healthy controls who underwent skin biopsy, there were no significant differences between groups in baseline characteristics, including age, sex, BMI, or smoking habits (S2 Table). Alcohol consumption was higher in controls compared to POTS patients. There were no significant differences in sensory thresholds measured with monofilament or neurothesiometry between groups (S2 Table). Median IENFD was comparable between POTS and controls (2.26 [1.62–3.08] fibers/mm vs. 1.63 [0.73–2.68] fibers/mm, respectively; p = 0.108) (Fig 1). We could not identify any obvious subgroup within POTS with low IENFD compared with healthy controls (Supplementary data file).

### 3.6. Measures of nerve function and IENFD in relation to self-reported neuropathy symptoms

Patients with POTS were divided into two groups according to their symptom status regarding peripheral neuropathy symptoms, based on the median value of the NSS in the whole group. Patients with NSS below 14.5 were included in the “Low NSS” group, and patients with NSS above 14.5 were included in the “High NSS” group. There were no differences in monofilament perception, neurothesiometer values, or IENFD between the two groups. Vibrometry Z-scores were higher in the “High NSS” group when measured in the little finger and foot, although the median values were still within normal ranges (Table 2). Six (31.6%) of the patients with an average vibrometry Z-score >1.65 in hands/feet were included in the “high NSS” group, whereas only one (5.3%) patient was included in the “low NSS” group (p = 0.090).

**Table 2:** Measures of nerve function in relation to self-reported neuropathy symptoms in POTS.

	Low NSS (<14.5) N = 21	High NSS (>14.5) N = 21	P-value
**Monofilament** Normal (%)			
Right foot	19 (95.0%)^a^	19 (95.0%)^a^	0.756
Left foot	20 (100%)^a^	19 (95.0%)^a^	0.500
**Neurothesiometer** (V)			
**Right side**			
Medial malleolus	7.0 (5.5–8.7)	7.5 (5.6–9.3)^a^	0.593
Big toe	4.8 (3.9–6.2)	5.3 (4.1–7.8)^a^	0.411
**Left side**			
Medial malleolus	5.7 (4.5–7.8)	7.4 (5.8–9.2)^a^	0.105
Big toe	4.7 (3.3–6.7)	5.6 (4.1–7.7)^a^	0.235
**IENFD (**fibers/mm)	2.40 (1.10–3.11)^a^	2.26 (1.66–2.85)^a^	0.930
**Multi-frequency vibrometry** (average Z-score)			
**Hand**			
Index finger	0.1 (−0.5–0.6)^b^	0.7 (−0.2–1.3)^b^	0.129
Little finger	0.0 (−0.5–0.8)^b^	0.9 (0.3–1.3)^a^	**0.006**
**Foot**	−0.3 (−0.6–0.4)^b^	0.6 (−0.1–1.2)^b^	**0.011**

Comparison of objective signs of peripheral neuropathy in patients with POTS with low and high burden of self-reported neuropathy symptoms measured with NSS [22]. Values are presented as number (percent) or median (interquartile range). Comparative analyses were performed with Fisher’s Exact test or Mann Whitney-U test. A p-value < 0.05 was considered statistically significant. NSS: Neuropathy Symptom Score; IENFD: Intraepidermal Nerve Fiber Density.

^a^1 missing value.

^b^2 missing values.

### 3.7. Measures of nerve function and IENFD in relation to self-reported gastrointestinal symptoms

Patients with POTS were divided into two groups according to gastrointestinal symptoms, based on the median value of the total IBS-SSS score. Patients with a total IBS-SSS score below 212.5 were included in the “Low IBS-SSS” group, and patients with total IBS-SSS score above 212.5 were included in the “High IBS-SSS” group. There were no differences in monofilament perception, neurothesiometer values, or IENFD between the two groups. Median vibrometry Z-scores were higher in the “High IBS-SSS” group when measured in the little finger and foot, however within normal ranges (Table 3).

**Table 3:** Measures of nerve function in relation to self-reported gastrointestinal symptoms in POTS.

	Low IBS-SSS N = 20	High IBS-SSS N = 20	P-value
**Monofilament** Normal (%)			
**Right foot**	20 (100%)	17 (94.4%)^b^	0.474
**Left foot**	20 (100%)	17 (94.4%)^b^	0.474
**Neurothesiometer** (V)			
**Right side**			
Medial malleolus	7.3 (5.4–9.2)	7.2 (5.7–8.7)^a^	0.593
Big toe	4.8 (3.6–6.8)	5.0 (4.3–6.8)^a^	0.411
**Left side**			
Medial malleolus	6.5 (4.7–8.3)	6.3 (5.2–7.8)^a^	0.105
Big toe	4.9 (3.3–6.7)	5.2 (4.0–6.5)^a^	0.235
**IENFD** (fibers/mm)	2.24 (1.21–3.16)^d^	2.26 (1.76–2.94)	0.942
**Multi-frequency vibrometry** (average Z-score)			
**Hand**			
Index finger	0.1 (−0.6–1.1)^c^	0.3 (−0.4–1.0)^a^	0.130
Little finger	0.4 (−0.7–0.8)^c^	0.7 (−0.1–1.3)^a^	**0.005**
**Foot**	−0.2 (−0.6–0.4)^c^	0.6 (−0.3–1.6)^a^	**0.010**
**Total NSS score**	11.0 (4.0–15.0)	18.0 (14.3–22.8)	**0.002**

Comparison of objective signs of peripheral neuropathy in patients with POTS with low and high burden of self-reported gastrointestinal symptoms measured with IBS-SSS [23]. Values are presented as number (percent) or median (interquartile range). Comparative analyses were performed with Fisher’s Exact test or Mann Whitney-U test. A p-value < 0.05 was considered statistically significant. NSS: Neuropathy Symptom Score [22]; IBS-SSS: Irritable Bowel Syndrome-Severity Scoring System; IENFD: Intraepidermal Nerve Fiber Density.

^a^1 missing value.

^b^2 missing values.

^c^3 missing values.

^d^4 missing values.

**Fig 2:**
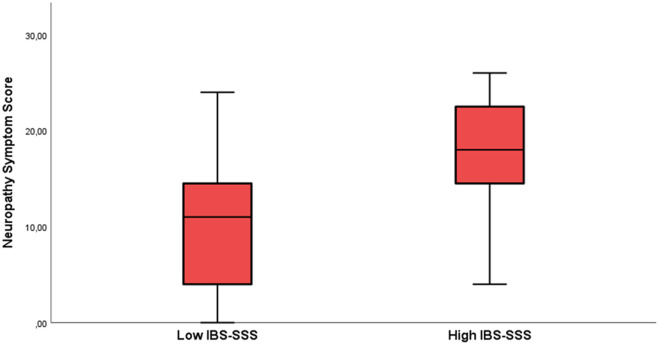
Comparison of neuropathy symptom scores in POTS patients with high or low IBS-SSS. NSS 11.0 (4.0–15.0) vs. 18.0 (14.3–22.8); p = 0.002. IBS-SSS: Irritable Bowel Syndrome-Severity Scoring System [23]; NSS: Neuropathy Symptom Score [22].

Total NSS was significantly higher in the “High IBS-SSS” group than in the “Low IBS-SSS” group (Table 3, Fig 2).

## 4. Discussion

The main finding of the present study was the absence of solid evidence for peripheral small or large fiber neuropathy in POTS when compared to healthy controls, despite a high burden of neuropathic symptoms. In addition, a high burden of gastrointestinal symptoms in POTS was not associated with neuropathy, based on objective measures. However, patients with a high burden of symptoms indicative of peripheral neuropathy had high scores on the IBS-SSS scale.

Questionnaires revealed that the neuropathy symptom burden is high in POTS, both in terms of occurrence and severity. Gastrointestinal symptoms in this POTS cohort are frequent and moderately severe, a finding published previously [15]. Regarding peripheral neuropathy assessed with NSS, the most commonly reported symptoms were abnormal sensations of heat and cold, which are symptoms we expected to find since heat and cold sensations are transmitted by small nerve fibers [17]. The patients also reported numbness and sensations of dull pain in both hands and feet, which are associated with dysfunction of moderately myelinated Aβ nerve fibers [17,29]. Our findings correspond to results from a large community-based cross-sectional survey in POTS patients, where coldness, tingling, and numbness in hands and feet were reported by 58–84% of the patients [6].

Neurological clinical status was fairly normal in patients with POTS in the present study, which is in line with previous studies on POTS where signs of neuropathy have been assessed [8,13]. A handful of patients had general muscle weakness, and thus, some unspecific neurological motor abnormalities were seen. Deconditioning and muscle weakness are common in POTS [1], probably due to orthostatic intolerance, which may explain these findings. We have previously shown that weekly physical activity is low in this POTS cohort [30].

Vibrometry Z-scores were abnormal in the hands and/or feet of seven POTS patients. When comparing the patients with high vs. low NSS, the patients with higher NSS had slightly higher Z-scores, measured on the little finger and the foot, than patients with fewer symptoms. Moreover, most of the patients with abnormal Z-scores reported high NSS. On the contrary, when measuring VPTs with neurothesiometry, all patients with POTS showed values within the normal age-adjusted range, and even somewhat lower values compared to the healthy control participants. No difference was seen regarding VPTs measured with neurothesiometry between patients with high vs. low NSS. This finding may suggest that MFV is a more sensitive instrument than the neurothesiometer. Unlike assessment with neurothesiometry, MFV measures VPTs for several different vibrating frequencies, which allows for a broader evaluation of large Aβ sensory nerve fibers, different mechanoreceptors, and different innervation areas [29]. The method is also less user-dependent than neurothesiometry [31]. Nevertheless, the observed differences were very small and remained within normal ranges in most patients. While these findings could indicate an early sign of neuropathy, it might also be coincidental and a multiple testing problem, and thus, we should be cautious in drawing any firm conclusions, given the very low values and small differences. In all, we did not find any solid associations between subjective symptoms of neuropathy obtained by questionnaires and clinical findings. This observation corresponds to the results from a recently published large study where no associations could be found between subjectively reported symptoms and objectively measured signs of dysautonomia in POTS [32].

It is estimated that approximately 50% of the POTS population have a neuropathic mechanism behind their symptoms [2], although no diagnostic criteria for neuropathic POTS exist [9]. This hypothesis has gained support from different assessment methods, including evidence of reduced IENFD on skin biopsies [7–10], findings of decreased norepinephrine spillover in the legs compared to the arms [3], supersensitivity of norepinephrine infusion in distal veins resembling the reaction in denervated veins [33], and impaired post-ganglionic sudomotor function upon measurement with QSART [9,12–14]. Theoretically, degeneration of small autonomic nerve fibers peripherally may lead to impaired vasoconstriction, causing blood pooling in the legs. To compensate for the decreased venous return, the body responds by increasing the heart rate [2]. In the present study, there was no significant difference in IENFD between POTS and controls. These findings are in contrast to previous studies on POTS, where reduced IENFD was found in 24–56% of the examined biopsy specimens [7–10,34,35]. In alignment with our findings, Singer et al. [36] did not find reduced IENFD in a small study of eight POTS patients. However, in three of the skin biopsy specimens, morphological abnormalities were found on the intraepidermal nerve endings [36]. The same study showed impaired post-ganglionic sudomotor function measured with QSART in POTS distally in the legs. This finding supports the thesis that autonomic nerve fiber dysfunction may be involved in the pathogenesis, rather than visible structural abnormalities [36].

The lack of solid association between subjective and objective neuropathic findings in POTS may be hypothetically explained by central sensitization, which has been described in IBS and other functional disorders of the gut-brain axis [37], as well as in other comorbidities commonly seen in POTS [38]. Additionally, somatic hypervigilance has been described in POTS [39,40]. Hence, central mechanisms may theoretically be the unifying link between self-reported symptoms of neuropathy and gastrointestinal symptoms in the present study.

The strength of the present study is that we have used a well-defined, quite large POTS cohort and a well-matched control material. Although the aim of the present study was not to perform a comprehensive testing battery of all types of peripheral somatic nerve fibers, we have utilized more easily accessible methods to scan for possible neuropathies that can explain the patients’ symptoms. We have explored symptoms and objective signs of both small and large fiber neuropathy with different modalities. All patients were examined clinically by the same physician (HT) to exclude other causes of neuropathy, and all participants filled in questionnaires regarding neuropathic and gastrointestinal symptoms in close temporal proximity to neuropathy testing. All MFV testings were performed by the same person (LE), all skin biopsies were obtained by the same surgeon (LD), and finally, all biopsies were examined by the same assessor (LE).

Regarding limitations of the study, the use of 5 µm thick sections of the skin biopsies is a limitation [41]. It is possible that this size is too small to find reduced IENFD in POTS. Skin punch biopsies with the assessment of IENFD in biopsy cuts of 50 µm are recommended for supporting the diagnosis of SFN affecting Aδ- or C-fibers and/or autonomous nerve fibers [41]. However, the assessment of 50 µm skin biopsies is time-consuming and not applicable to clinical routine [4]. We have recently demonstrated that 5 µm biopsy sections can be used for the assessment of IENFD in diabetic subjects with reliable results [28]. A similar, clinically oriented method for assessment of IENFD has been used in Finland with 10 µm skin biopsy cuts, also with reliable results [42]. Further limitations include that we did not have access to data on heart rate variability and other variables reflecting cardiac autonomic reflex tests [9,43]. It is possible that a subgroup with abnormal cardiac autonomic reflex tests would present with objective signs of neuropathy, including reduced IENFD, according to previous studies [9]. However, in the present cohort, no obvious subgroup with low IENFD was identified. Furthermore, aspects of cardiovascular function in POTS are already well described, hence the focus of the present study to examine peripheral nerves and associations between peripheral neuropathy and gastrointestinal symptoms in POTS. Another potential limitation was that the sample size was too small to explore associations between various symptoms and objective findings within the POTS cohort. We did have a rather small control group for comparisons regarding IENFD, and it is difficult to find healthy controls who are willing to go through an invasive procedure such as a skin punch biopsy. Yet, all control participants were well-matched with the POTS patients, and the results from our study are comparable to normative IENFD values obtained from the same laboratory and the same hospital [27]. There is also a risk of selection bias since the patients were recruited from a highly specialized syncope center, and the patients who agreed to participate in the study might have suffered more from symptoms indicative of peripheral neuropathy than the general POTS population. Other limitations include that the patients continued to take their regular medications during the study, and finally, the many co-morbidities that patients reported.

For future perspectives, it would have been interesting to add measurements of peripheral small nerve fiber function, e.g., with QSART test, to explore potential associations to symptoms of peripheral neuropathy. However, QSART is not available at our hospital, requires both highly trained staff and sensitive equipment, and is thus most often used within specialized centers [11].

## 5. Conclusion

Symptoms indicative of peripheral small and large nerve fiber neuropathy are common in POTS and are associated with a high burden of gastrointestinal symptoms. However, no solid evidence of peripheral small or large fiber neuropathy was found in POTS when compared to healthy controls in this cross-sectional study, based on objective measures. Future studies are needed to further evaluate the potential presence of small fiber neuropathy in POTS and other conditions of autonomic neuropathy.

## Supporting information

S1 Fig Flow chart demonstrating the recruitment process of POTS patients. (TIF)

S1 Table Neuropathy Symptom Score categories, and severity of symptoms within healthy control participants and patients with POTS. (DOCX)

S2 Table Clinical characteristics and subjective and objective signs of sensory nerve fiber neuropathy in control participants and patients with POTS who performed skin punch biopsy. (DOCX)

S1 File Data on symptoms and objective signs of peripheral neuropathy. (XLSX)
