# Supplementary figures and images for: Symptoms and objective signs of peripheral sensory neuropathy in POTS and correlations to gastrointestinal symptoms
Source: PLoS One. 2025 Jul 3;20(7):e0327549. doi: 10.1371/journal.pone.0327549 (PMC12225795; doi:10.1371/journal.pone.0327549)

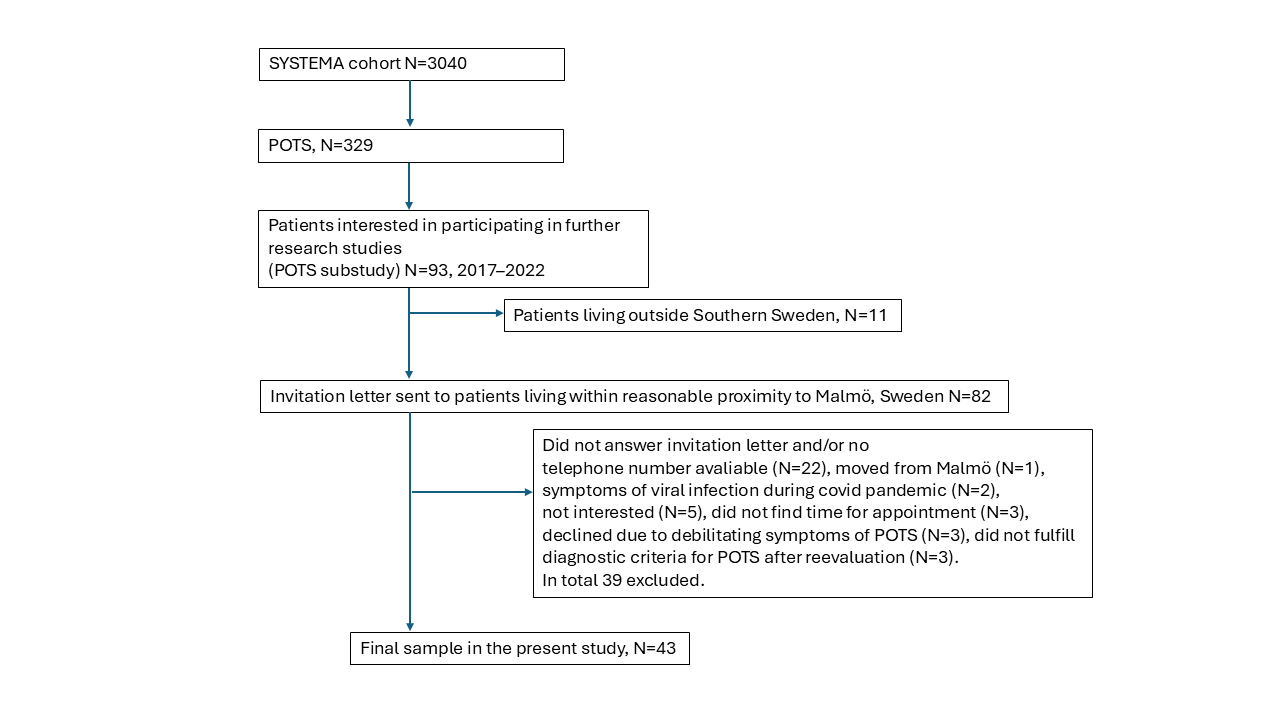

Supplement: S1 Fig — (TIF) [file pone.0327549.s001.tif]
