# Supplementary material for: Symptoms and objective signs of peripheral sensory neuropathy in POTS and correlations to gastrointestinal symptoms
Source: PLoS One. 2025 Jul 3;20(7):e0327549. doi: 10.1371/journal.pone.0327549 (PMC12225795; doi:10.1371/journal.pone.0327549)
Supplement: S1 Table — (DOCX) [file pone.0327549.s002.docx]

**S1 Table. Neuropathy Symptom Score categories and severity of symptoms within healthy control participants and patients with POTS**

|  | **Controls,** N = 50^b^ |  | **Patients,** N = 42^a^ |  |
| --- | --- | --- | --- | --- |
|  | **Hands** | **Feet** | **Hands** | **Feet** |
| **1. Numbness** |  |  |  |  |
| Never | 38 | 48 | 14 | 14 |
| Sometimes | 10 | 1 | 13 | 13 |
| Often |  | 1 | 10 | 15 |
| Nocturnal | 2 |  | 5 | 1 |
| **2. Abnormal sensation to heat or cold** |  |  |  |  |
| Never | 44 | 42 | 9 | 7 |
| Sometimes | 6 | 7 | 9 | 8 |
| Often |  |  | 24 | 25 |
| Nocturnal |  | 1 | 1 | 3 |
| **3. Sensation of pins and needles** |  |  |  |  |
| Never | 38 | 49 | 11 | 8 |
| Sometimes | 9 | 1 | 12 | 16 |
| Often | 1 |  | 17 | 18 |
| Nocturnal | 2 |  | 3 | 1 |
| **4. Burning pain** |  |  |  |  |
| Never | 50 | 50 | 27 | 23 |
| Sometimes |  |  | 9 | 11 |
| Often |  |  | 6 | 7 |
| Nocturnal |  |  | 1 | 2 |
| **5. Lancinating pain** |  |  |  |  |
| Never | 50 | 50 | 20 | 19 |
| Sometimes |  |  | 12 | 15 |
| Often |  |  | 11 | 9 |
| Nocturnal |  |  |  |  |
| **6. Dull pain** |  |  |  |  |
| Never | 48 | 50 | 16 | 18 |
| Sometimes | 2 |  | 10 | 8 |
| Often |  |  | 17 | 17 |
| Nocturnal |  |  |  |  |
| **7. Contact dysesthesia to bedclothes** |  |  |  |  |
| Never | 50 | 49 | 31 | 30 |
| Sometimes |  | 1 | 4 | 5 |
| Often |  |  | 2 | 1 |
| Nocturnal |  |  | 5 | 6 |

Number of participants within each categorization and severity of the NSS questionnaire

^a^1 missing value.

^b^4 missing values.
