# Supplementary material for: Symptoms and objective signs of peripheral sensory neuropathy in POTS and correlations to gastrointestinal symptoms
Source: PLoS One. 2025 Jul 3;20(7):e0327549. doi: 10.1371/journal.pone.0327549 (PMC12225795; doi:10.1371/journal.pone.0327549)
Supplement: S2 Table — (DOCX) [file pone.0327549.s003.docx]

**S2 Table**. **Clinical characteristics and subjective and objective signs of sensory nerve fiber neuropathy in control participants and patients with POTS who performed skin punch biopsy**

|  | **POTS**  (N=39) | **Controls**  (N=13) | **P-value** |
| --- | --- | --- | --- |
| **Age** (years) | 30.6 (27.0–41.0) | 29.3 (36.3–40.5) | 0.071 |
| **Female** (%) | 36 (92.3 %) | 11 (84.6 %) | 0.589 |
| **BMI** (kg/m^2^) | 24.4 (21.3–27.2) | 21.7 (20.6–24.0) | 0.142 |
| **Never smoked** | 29 (74.4 %) | 9 (69.2 %) | 0.777 |
| **Alcohol consumption less than 1 glass/week** | 35 (87.2) | 1 (7.7) | **<0.001** |
| **Total IBS-SSS** | 245 (126–321) | 8 (0–41) | **<0.001** |
| **Total NSS** | 15 (6–22) | 0 (0–0) | **<0.001** |
| **Neurothesiometer** (V) |  |  |  |
| **Right big toe** | 4.9 (4.0–7.4) | 5.1 (4.2–7.2)^a^ | 0.691 |
| **Left big toe** | 5.0 (3.3–6.7) | 4.5 (3.8–7.3)^a^ | 0.829 |
| **Right malleolus** | 7.3 (5.5–8.8) | 6.2 (5.1–7.5) | 0.340 |
| **Left malleolus** | 6.5 (4.7–8.0) | 6.8 (5.6–7.7) | 0.856 |
| **IENFD** (fibers/mm) | 2.26 (1.62–3.08) | 1.63 (0.73–2.68) | 0.108 |

Values presented as numbers (percent) or medians (interquartile range). Comparative analyses were performed with the Fisher’s Exact test, Chi2-test, or Mann-Whitney U test. A p-value < 0.05 was considered statistically significant. IBS-SSS: Irritable Bowel Syndrome – Severity Scoring System; NSS: Neuropathy Symptom Score; IENFD: Intraepidermal Nerve Fiber Density

^a^1 missing value.
